# Conjugation of Nanomaterials and Nematic Liquid Crystals for Futuristic Applications and Biosensors

**DOI:** 10.3390/bios8030069

**Published:** 2018-07-14

**Authors:** Amit Choudhary, Thomas F. George, Guoqiang Li

**Affiliations:** 1 Department of Physics, Deshbandhu College, University of Delhi, Kalkaji, New Delhi 110019, India; amitnpl2005@gmail.com; 2 Departments of Chemistry & Biochemistry and Physics & Astronomy, University of Missouri–St. Louis, St. Louis, MO 63121, USA; tfgeorge@umsl.edu; 3 Visual and Biomedical Optics Lab, The Ohio State University, Columbus, OH 43212, USA; 4 Biomolecular Sciences Institute, Florida International University, Miami, FL 33199, USA

**Keywords:** nematic liquid crystals, gold nanoparticles, biosensors, liquid crystal biosensors

## Abstract

The established role of nematic liquid crystals (NLCs) in the recent rapid development of displays has motivated researchers to modulate the electro-optical properties of LCs. Furthermore, adding nanomaterials into NLCs has led to enhancements of the properties of NLCs, like reduced threshold of the operating voltage, variation in pretilt angle, reduced switching time, etc. These enhanced properties, due to interfacial dynamics, are enabling wider applications of NLCs and nanomaterials. The recent literature of nanomaterial-doped NLCs is rich with various kinds of nanomaterials in a variety of NLCs. The light has been focused on the most widely used and studied gold nanoparticles in NLCs. The intrinsic inherent property of easy excitation of surface plasmons polaritons (SPP) is the mediating interaction of NLC electric dipoles and the polarization of charges in the GNP surface. The concepts and methods for the application of metal nanomaterials as dopants in NLCs are discussed for future applications, especially biosensors. The biosensing application of NLCs alone has already been proven in the literature. However, it is always desirable to further enhance the detection efficiency and selectivity, which have been achieved by the conjugation of GNPs and nickel nanoparticles with NLCs and their compatibility with biological materials. This aspect of future application of nanoparticles and NLC makes the point more selective to be included in the present manuscript.

## 1. Introduction

Nematic liquid crystals (NLCs) have a rich literature about their fundamental and practical applications [1,2,3]. Adding nanomaterials (metallic and non-metallic) to NLCs has facilitated improving electro-optical properties and augmenting the functions of NLC [3,4,5,6,7]. A variety of nanoparticles have been used to further enhance the fundamental properties of NLCs, such as ferroelectric nanoparticles being used for increasing the dielectric anisotropy [8,9]. Multiferroic bismuth ferrite has been doped to achieve a superior electro-optic response of NLC devices [6,7,8]. Nanoparticles (NPs) of metals, such as Au [4,10] and Ag [11], have also led to a modification in the elastic properties and rotational viscosity of NLC composites.

The addition of an external agent, particularly particles, has shown the rearrangement of the NLC molecules around the particles. At the microscopic scale, adding external entities (spherical microparticles) in NLCs has shown rearrangement in the alignment of NLC molecules around entities to form various possible structures. Such structures could be like Saturn rings, boojams, and hedghogs, which have been studied both theoretically and experimentally, showing the dependence on their size and shape [12,13,14,15,16,17,18,19,20,21,22,23,24,25,26,27,28,29,30,31,32,33,34,35,36,37,38,39,40,41,42,43]. The anisotropic molecular alignment around gold nanoparticles (GNPs) has been verified in experimental observations of NLC materials [44]. The addition of GNPs in NLCs has shown a remarkable red shift of the surface plasmon polaritons (SPP) peak in the absorption spectra of NLC/GNP composite systems due to an increment in the refractive index of an NLC when light is polarized parallel to the director of the NLC [45]. The shift in the SPP peak is highly dependent on the dielectric constant close to the interface of the GNP and NLC. This is due to the fact that the electron cloud in the GNP is highly influenced by the dielectric environment.

On the other hand, NLCs have attracted attention towards a label-free biological detector for various biomolecules and viruses. Chemical and biological detection using NLCs is opening a new dimension for the application of NLCs due to their dynamics of tunable interfacial molecular alignment [46,47,48,49,50].

Introducing nanoparticles (metallic and non-metallic) in NLCs has created a targeted source of detection for certain biomolecules/viruses [46,48,51,52]. Nanomaterials like gold, silver, CdS, carbon nanotubes, quantum dots, etc. have been used as dopants for the purpose of improving the properties of NLCs. The application of metallic and semiconductor nanoparticles has improved the intrinsic properties of NLCs for their applications in both displays and non-displays. It should be noted that in general NLC materials have been applied for sensing of cancer, cholesterol, glucose, gas in environment, pH, temperature, thrombin, protein, etc. In the present article, we focus on metallic nanoparticles as dopants and their application in NLCs. Nanoparticle-doped NLCs have shown potential for detecting the target in biosensing applications.

**Figure 1:**
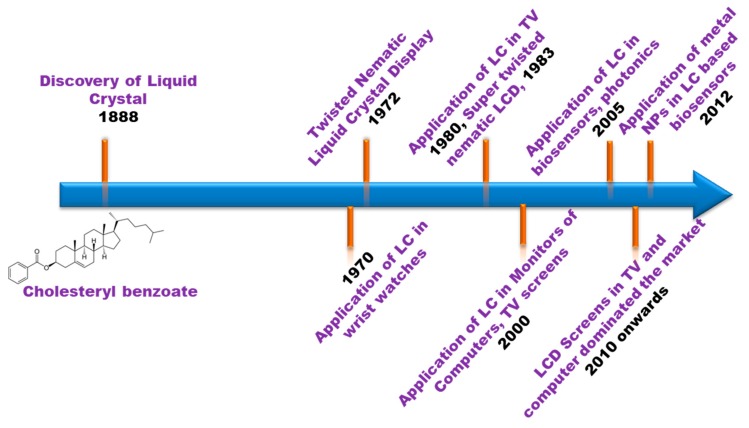
Schematic of significant developments in the history of liquid crystals. As indicated, the first display took around hundred years for the commercialization of LC displays (LCDs) after the discovery of liquid crystals.

Since NLCs are very sensitive to electric and magnetic fields, solid surfaces and any other external agents, they have been used for a variety of applications in the last several decades, as shown in Figure 1. In this regard, NLCs are suitable materials for displays [53,54], non-displays such as adaptive lenses and filters [55,56,57,58,59,60], and sensors, particularly biosensors [51]. There are some basic concepts for NLC based biosensors. This is interfacial dynamics of NLC molecules with a solid substrate mediated by anti-body/antigens or simply biomolecules that allow the variation in the signaling of biomolecular presence. The NLC molecules are well aligned at the interface of reference, but when the surface of interference is loaded with biomolecules, the molecules show distortions, which result in blurred transmission under crossed polarizes of a polarizing microscope and reflects the changes in texture. In other words, the change in the molecular structure due to orientation of molecular director at biomolecule-NLC interface is the result of the interaction of biomolecules with NLC which triggers the reorientation of director from one configuration to another. This transition could extend up to a few micrometers in depth of bulk NLC from the interface. This information of change due to reorientation of molecular director can be easily transduced by crossed polarizers of polarizing optical microscope and sometimes by electrical methods as well [61].

This present review is not intended to provide a full overview of the wide rich works of published literature in the field of nanoparticles and NLCs, since this could be obtained elsewhere in various review articles [4,62,63]. Rather, we provide an analysis of the current literature on the application of metallic nanomaterials in NLC and their applications, particularly as biosensors, in regard to futuristic technology. The metal nanoparticles have the advantage of tuning the SPP if the environment in the close proximity is changed. Such changes in the SPP are a key method of analysis for any interaction formed by some kind of assembly of molecules. A gold nanoparticle is a suitable candidate for this purpose and hence chosen for the analysis in the present review. In the case of NLCs, it has been observed that the rearrangement of NLC molecules around GNP are defined in the form of defects but the observation of such a defect at the nanoscale is difficult under the crossed polarizers of optical polarizing microscope, needing further experiments and analysis at nanoscale. Since the SPP mediated interaction between GNP and NLC/any other medium leads to the key changes in the properties of the host medium, GNP and NLC systems could be one of the appropriate ways for the application of NLC as a biosensor. The addition of nanoparticles in the NLC increases their applicability for the selectivity of biomolecules and signal/observation enhancement. However, the biomolecular sensing by an NLC requires further research, particularly for understanding biomolecular interactions of the NLC and biomolecules which could give fast and reliable sensing of various biomolecules. In addition, the mediation of nanoparticles for biosensing between the NLC and biomolecules also requires rigorous studies.

NLCs have a long history of their development. The schematic diagram in Figure 1 shows the timeline of the development of LC from the discovery to the present days where LC are also being commercialized for various applications other than mere displays, optical modulations in optical communications, optically addressable spatial light modulators (OSLMs), electronically addressable spatial light modulators (ESLMs), and more. The development of LCs has proven the significance of intense work of researchers for understanding the science of LC from molecular to physical level and their possible utilities in various applications. The present review has been categorized into the following sections: materials and methods, which describe the method of sample cell preparation for different purposes; and results and discussion, where the results considered under the present review are analyzed and the bio-sensing using GNP and NLC aspect is discussed.

## 2. Materials and Methods

Fabrication of Liquid Crystal Sample Cells

The most important part of NLC-based devices is the sample cell fabrication. A particular type of sample is necessary for specific displays, fundamental studies with electro-optical and dielectric spectroscopy, biological detections, etc. Sample cell fabrications for selected purposes are reviewed below:

a. **Sample Cell Preparation for NLC Electro-Optical Studies:**

Here the NLC materials are basically studied with polarizing optical microscopy for their electro-optical properties. The material is sandwiched between two transparent electrodes. The conducting electrodes are designed using a transparent indium-tin-oxide (ITO) thin film deposited on a glass substrate. A desired pattern of the ITO electrode can be carved using photolithography techniques [44,55,56,57,58,64]. In order to achieve alignment of the NLC molecules, one needs to pre-treat these electrodes with appropriate chemicals. These chemicals could be nylon 6or polyvinyl alcohol (PVA) for homogeneous alignment and silane, SE 1211 polymer, etc. for vertical alignments of NLC molecular director [44]. The two electrodes are assembled in the form of a capacitor. The NLC material is sandwiched at an elevated temperature of NLC so that it can reach in between the electrodes by means of capillary action.

b. **Sample Cell Preparation for Nanoparticle-Doped NLCs:**

The nanoparticles are mixed in an NLC first manually, and then kept in an ultrasonic bath for 30 min to 1 h for their mixing properly by maintaining a suitable temperature of the ultrasonic bath. If the nanoparticles are in a liquid solvent, then the solvent needs to be evaporated at a fixed temperature, so that no traces of solvent can be found in the NLC/nanoparticle composite. Then, the NLC/nanocomposite composite material is sandwiched in the sample cell by means of capillary action at an elevated temperature for around 30 min, and after that the cell needs to be cooled down naturally or by controlling the cooling rate to room temperature.

c. **Sample Cell Preparation for Biological Detections Using Nanoparticles and NLCs:**

The glass substrate is cleaned with an aqueous solution of H_2_O_2_, and sometimes with a H_2_SO_4_ solution as well [65,66,67,68]. As per some reports, the biomolecules are first loaded on well-cleaned glass substrates which are assembled to form the sample cells, and then the NLC is filled in the sample cell at the isotropic phase by means of capillary action. The NLC alignment is noticed under a polarizing optical microscope and by measuring its impedance. The sensor so formed is calibrated with various concentrations. In another biomolecular sensing mode, the antigen and antibody are dispersed in the aqueous phase. Then the NLC is arranged to make an interface with this aqueous phase, and the NLC molecules undergo reorientation at the interface of the NLC and aqueous phase [69], using the interface of nanoparticles [48].

In order to enhance the sensitivity and selectivity of detection, nanoparticles are added to the NLC and biological systems in which the homeotropic alignment is achieved by nickel nanoparticles. Then aptamer functionalized GNPs at the surface cause a disruption of the NLC alignment [70].

In the same manner as the samples are prepared for different purposes, the mode of operation is also different. In the display mode, the birefringence is changed by applying the electric field, and the resulting transmission is used for the transfer of information. On the other hand, in biological detections, the birefringence is changed spontaneously as the NLC molecules are anchored with biomolecules, and this is detected in the transmission of the sample cell.

## 3. Results and Discussion

### 3.1. Nematic Liquid Crystal Molecular Alignment at the Interface of Nanoparticles

**Figure 2:**
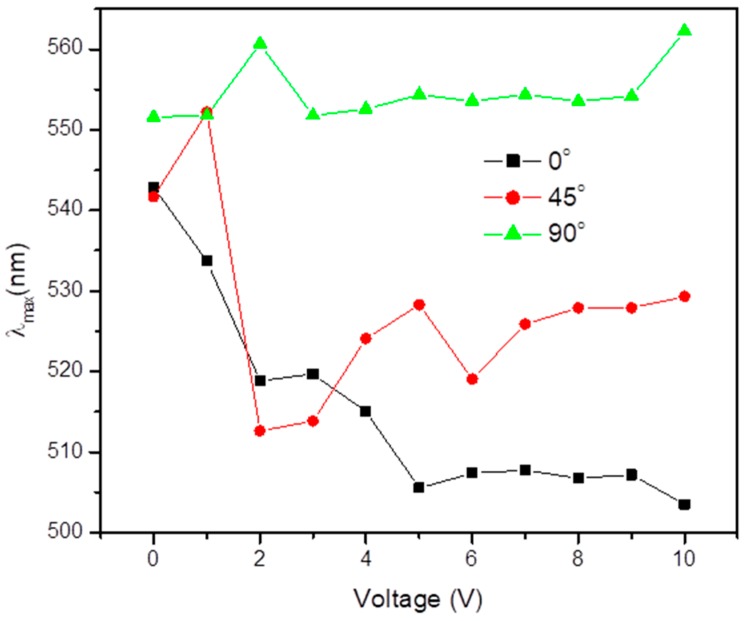
Optical absorption peak wavelength against a wide voltage range of an external applied electric field at 0°, 45° and 90° angles between the incident polarized light and the rubbing direction. Reproduced with permission from Ref. [44]. (Optical Society of America).

Studies of the alignment at the interface of nanoparticles are significant for the properties of NLC systems, their functioning and characterization. Various topological defects have been proposed around the colloidal particles (micrometer size) [31,37,38,39,40,41,42,43,69,70,71,72]. However, visualization of this kind of alignment around nanoparticles in optical textures under a microscope is challenging. Optical studies by UV-visible spectroscopy have shown anisotropic shifting of SPP peaks in the absorption spectra when there is a change in the plane of polarization of light incident on the sample with respect to the rubbing direction of the NLC sample cell [44,45,73]. As shown in Figure 2, the alignment of the NLC sample cell is vertical (homeotropic) and does not show any shift in the SPP peak at 0 V with respect to change in the angle of plane of polarization of incident light. Also, the vertical alignment was established under crossed polarizers of polarizing microscope by confirming the dark state of the material. Now in order to analyze whether there is any material or this state is due to non-availability of NLC, the bright state was achieved by applying the dc bias across the ITO electrode. This confirms the vertical alignment corresponding to the dark state of NLC cell [44]. But when the alignment is switched from vertical to planar by a DC bias voltage of 6 V, one sees a shift in the absorption spectra due to the anisotropic change in the dielectric constant around GNPs [44]. Pratibha et al. have also observed a red shift in SPP peak from 0° to 90° in the polarization [45,73].

**Figure 3:**
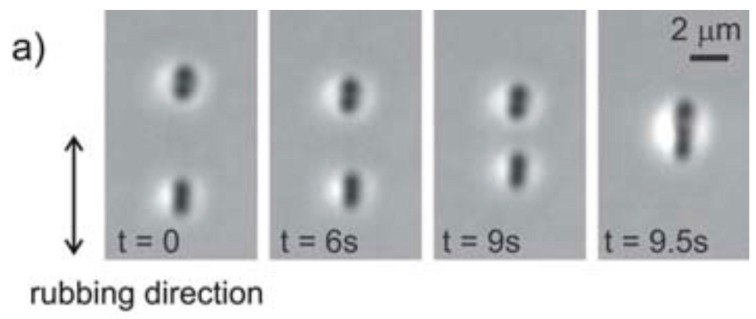

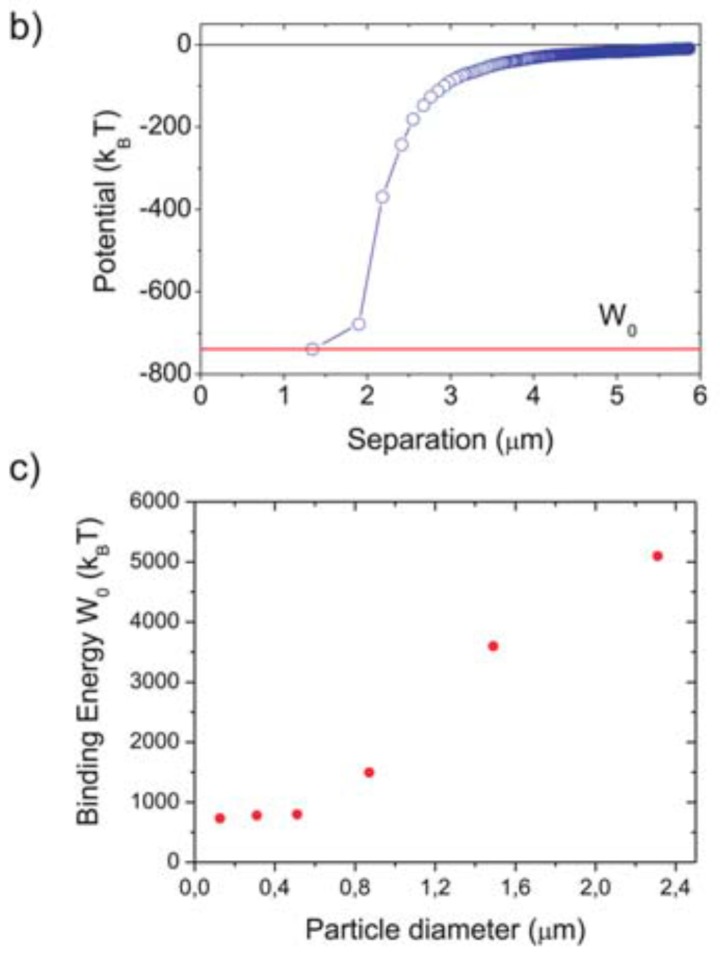
(**a**) Two dipolar nanoparticles of 125 nm in diameter are attracted along the nematic director into the dipolar pair. (**b**) The separation dependence of the attractive potential and the minimum of the attractive potential representing the binding energy W_0_. (**c**) The measured binding energy of two dipolar particles is decreased by reducing the size of the particles, but is approximately constant below 500 nm. Reproduced with permission from Ref. [78]. (Copyright 2010, Royal Society of Chemistry).

Alignment around NPs can be varied using external stimuli [74]. The other approach used for the modification in the alignment is through the self-assembly of GNPs using the Ostwald ripening process [75,76,77]. The hyperbolic defects created by alignment of NLC molecules around nanoparticles make the particles look much bigger than their actual size. Such a hyperbolic defect is sometimes called a defect dipole due to its arrangement with the defect and particle, analogous to an electric dipole, as shown in Figure 3. This arrangement of particle and defect exerts a force of attraction between two such nanoparticles pairs. This is dependent on the size of the particles and the distance between the particle and defect, along with the separation between the pairs. Also, the force is dependent on the alignment direction of the sample cell [78]. The NLC molecular arrangement plays a very crucial role in self-assembly of the nanoparticles and the associated molecular dynamics in nematic colloids systems. The long-range control of NP over the NLC alignment due to surface interaction of NP leads to the assembly of stable structures and superstructures of NPs. Such structures have the potential to be employed in certain applications like periodic photonic structure, biosensing, etc.

### 3.2. Self-Assembly of Nanoparticles through Nematic Liquid Crystals

**Figure 4:**
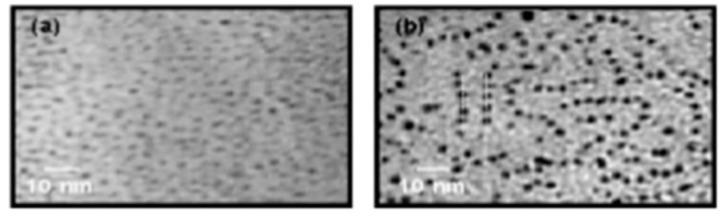
Transmission electron microscopy (TEM) images of Au nanoparticles with NLC ligands (**a**) before and (**b**) after thermal treatment. Reproduced with permission from Ref. [79]. (Royal Society of Chemistry).

**Figure 5:**
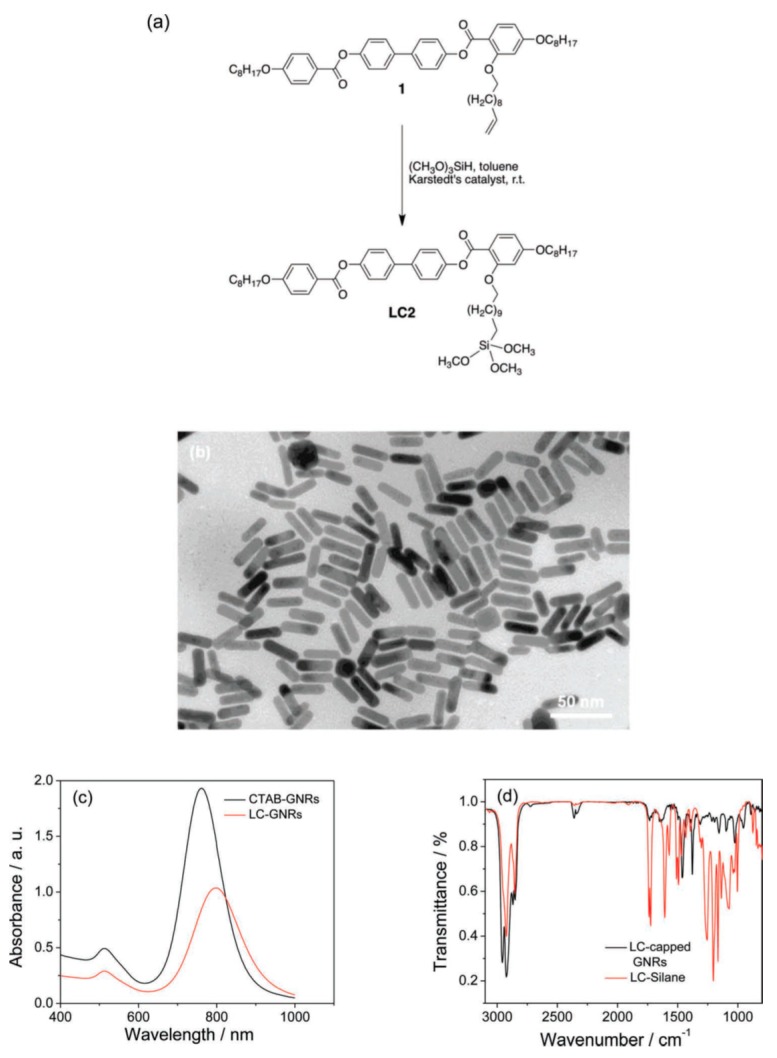
(**a**) Synthesis of the LC silane LC2. (**b**) TEM image obtained for CTAB-stabilized gold nanorods (GNRs) in water. (**c**) UV-visible-NIR spectra recorded for CTAB-capped GNRs. (**d**) Overlapping IR spectra of the capped GNRs and LC silane. Reproduced with permission from Ref. [80] (WILEY-VCH Verlag GmbH and Co. KGaA, Weinheim, Germany).

Self-assembly of nanoparticles in an NLC is an important part of research for nanoparticle-based LC devices, besides the individual nanostructural properties of the nanoparticles. The self-assembly is based on the mediator molecules between the NLC and nanoparticles. Such types of molecules are like thiol ligands that are a common trend to produce vertical alignment of an NLC on the surface of nanoparticles [4]. Kim et al. [79] observed one-dimensional self-assembly of spherical GNPs in the NLC system (Figure 4). The gold nanorods show the self-assembly structure in NLC taking advantage of their anisotropic shape along with the anisotropic nature of NLC materials (Figure 5) [80].

**Figure 6:**
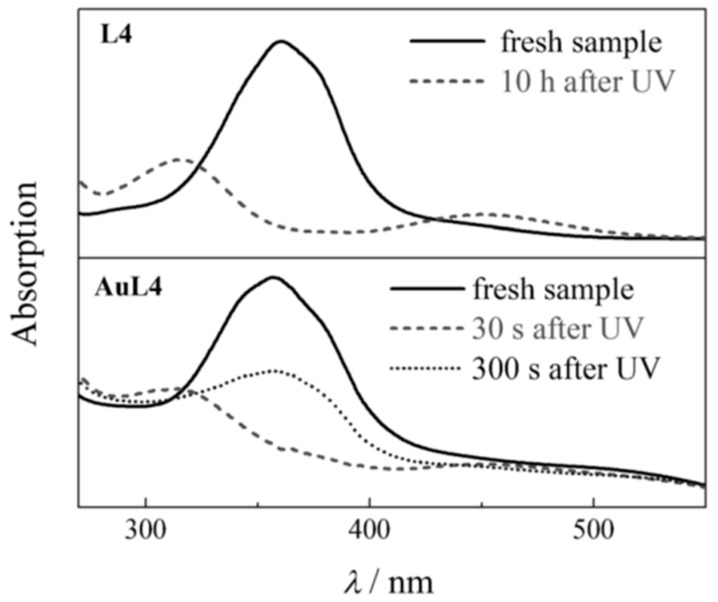
UV-visible spectra for ligand L4 (c = 10^−6^ M) dissolved in chloroform before UV irradiation (solid line) and 10 h after the UV light is turned off (dashed line, top graph); and for GNPs AuL4 dissolved in chloroform before irradiation (solid line), 30 s after the UV light is turned off (dashed line), and 300 s after the UV light is turned off (dotted line, bottom graph). Reproduced with permission from Ref. [81]. (John Wiley & Sons).

Another aspect of mediating molecules between GNPs and NLC is the coating of a photosensitive azo dye in large density at the surface, which makes the GNP/NLC system a photo (UV) tunable system in which the LC structure can be melted, but the LC structure can be recovered by switching off the UV light. The phase transformation from the LC phase to an isotropic one is interpreted as isomerization of azo units from the trans to the cis conformations (Figure 6) [81].

On the other hand, the coating of LC molecules via thiol ligands on a GNP exhibits the 3-dimnesional superlattice structure constituted by GNPs, which presents the varying lattice parameters with an increase in the number of nanoparticles in a cluster, keeping the ligand constant [82]. Such a concept of molecular attachment of an NLC with other NPs needs an investigation of the anchoring energy that is responsible for various types of self-assemblies of nanomaterials in an NLC matrix. There are different parameters responsible for the anchoring of NLC molecules on the NPs, such as the anchoring energy on the surface of an NP and the size of the particles. The anchoring energy plays a very important role in aligning the NLC molecules as vertical or planar to the surfaces of NPs. Assuming the anchoring energy w = WR/K = R/a, where K is the elastic constant, W is the surface anchoring coefficient, R is the radius of the NP, and a is the surface exploration length (K/W) [83,84]. If w > 1, then the alignment is expected to be planar; and if w < 1, then it is perpendicular to the surface of the NP. This is valid for a large radius ratio of the particle to the NLC molecule. For a particle with radius comparable to the size of the molecules, i.e., at the nanoscale, the anchoring energy is w = 1. W is kept in the range of 10^−6^–10^−4^ J/m^2^ and K around 10^−11^ J/m. The radius of the particle ranges from 100 nm to 10 µm. That means if the radius is taken as smaller than 100 nm, w would be smaller than 1, resulting in a homeotropic alignment of NLC molecules on the surfaces of the NPs [84,85].

The sputtering technique for dispersion of Au NPs into an NLC has also been developed in which sputtered Au atoms from the target are evaporated over the surface of the NLC [86]. Since the Au atoms are smaller than the size of the NLC, the Au NPs penetrate into the NLC without segregation. As the exposure is continued, segregation starts to form Au at the nanoscale in the NLC. The particles could attain a particular size of NP due to attachment of NLC molecules around the GNPs.

**Figure 7:**
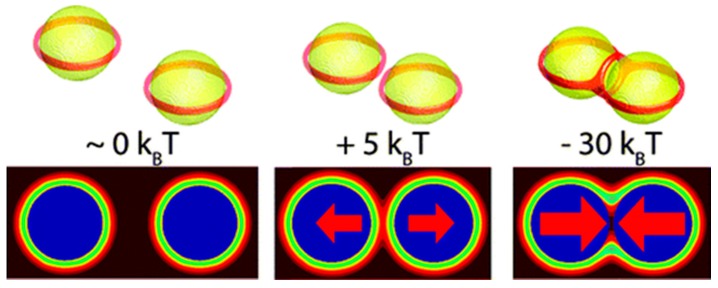
Three-dimensional representation of the defect structure and contour plot of the scalar order parameter S along the horizontal plane passing through the center of two nanoparticles, with R = 25 nm and strong anchoring with W = 10 mJ/m^2^. (**a**) Distance between particles of r = 80 nm, (**b**) r = 40 nm and (**c**) r = 10 nm. Notice that once the separation between particles is the same order of magnitude as the nematic-coherence length, the defects interact to form a three-ring structure. Reproduced with permission from Ref. [87]. (American Chemical Society).

The two cases of anchoring conditions (strong anchoring W = 10 mJ/m^2^ and weak anchoring W = 0.3 mJ/m^2^) have shown the impact on the molecular alignment of NLCs around NPs. For strong anchoring, a Saturn ring-type of defect structure situated away from the NP surface has been proposed based on theoretical studies. For weaker anchoring of NLC on the NP surface, the ring is observed with a stripe-like region over the surface. When the nanoparticles are well separated, the Saturn rings are formed about each NP of radius around 25 nm, whereas when they approach each other, a three-ring structure defect is formed, which could play a crucial role in understanding the dynamics of molecular structuring in the self-assembly process of NPs in NLCs, as shown in Figure 7 [87]. The saturn ring structure around the NPs would have different energy than the rest of NLCs, which could play the key role in binding the external agent close to the NPs and can be helpful in making a device.

There are certain advantages of using metal NPs as discussed in the literature and in the current review. This can be easily realized through the fact that the metal NPs have improved the properties of NLCs resulting in the increase in the pretitl angle due to the modification of the elastic properties, lower operating voltage, reduced Frederick transition threshold, etc. Undoubtedly, the size of NPs is very significant due to the change in their own properties as compared with the bulk, i.e., quantum confinement. The quantum level interaction of NPs with NLC is still unexplored and need rigourous work to be carried out for understanding the exact interaction between the two elements. Since the size of the NPs is comparable to the molecular size of NLC, so the tiny distortion in alignment of the NLCs does not change the optical transmission obviously. On the other hand, the new properties are also realized, such as striped pattern of GNPs, nano antenna, self-assembled chain of GNPs, etc., which could be employed for designing miniature devices at the micro/nano scale.

Along with the advantages, there could be some disadvantages as well. The uncontrolled doping of NP could disturb the uniform alignment of NLCs. The increase in the conductivity due to the addition of ions as impurity or charged NPs in excess could lead to the heating effect of the device and hence o the reduction in the performance of the device.

### 3.3. Concept of Biosensing Using Nematic Liquid Crystalsvia Nanoparticles

**Figure 8:**
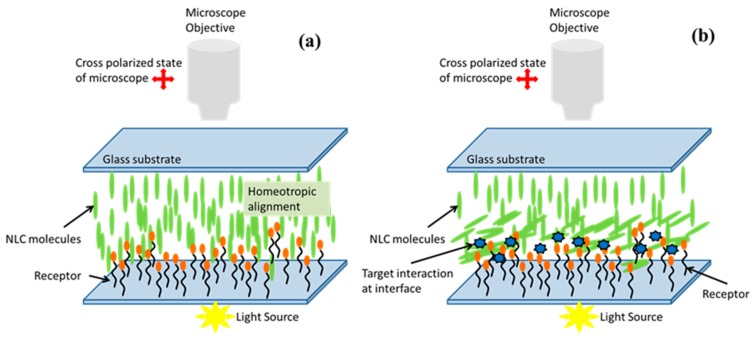
Schematic diagram of one of the concepts used in the biosensing by NLCs. (**a**) Only the receptor on the substrate is able to induce the homeotropic alignment of NLCs, (**b**) The NPs are introduced along with the receptor with required specification that disrupts the molecular alignment of the NLCs and change in transmission is observed under polarizing microscope.

In recent trends, the expanding applications of NLCs in various disciplines other than displays include photonics and biological detections. The application of NLCs in biological detection is through the observation of various parameters like impedance, capacitance and change in optical texture of the sample via spontaneous change in transmission and detected through crossed polarizers of a polarizing microscope caused by reorientation of NLC molecules, as represented in the schematic Figure 8. In recent years, the biological sensing utilizing NLCs has become an interesting topic for applied and basic research on the understanding of molecular interactions of biomolecules with an NLC matrix [88,89,90]. The interfacial functionality of NLC has made it a feasible material for detection of biological molecules, such as proteins, oligopeptides and nucleic acids, displayed on the surfaces of solids [91,92,93,94]. The NPs could be used for biosening by either deposition on the substrate before NLC interaction or by insertion into the NLC system or both for signal enhancement. Correspondingly, in one type of biosensor, NPs are deposited on the substrate along with specific biomolecules before the filling of the NLC in the sample cell, and in another type, the mixture of the NPs and the biomolecules is inserted into the NLC [53,70,95,96].

In order to enhance the output signal, continuous efforts are being made by employing metallic nanoparticles as mediator for the interaction of biomolecules and LC molecules, so that the detection could be transduced efficiently and reproducibly for the reliable device fabrication [48,70,95,96,97,98].

It is well known that there are certain parameters that are capable of shifting the SPP peak [99]. The resonance frequency of SPP in metal NPs is well known to be dependent on their shape, size, material properties (dielectric, optical, etc.), and surrounding medium. The SPP is a wave of oscillation of electron cloud near the particle surface. This wave of electron cloud originates from a strong electric dipole having a strong electric field close to the surface of the NPs and then in the bulk. This dipole is supposed to interact with other entities also, which make the metal NPs different from non-conducting NPs. The surface plasmons are well known for nickel NPs and other metal NPs which can be utilized for better biosensing with high signal-to-noise ratio [100]. Recently, Nickel NPs have been employed along with NLCs for biosensing applications [70,96]. The defect formation around the NPs in the form of elastic dipoles could be capable of binding the biological entities through NLC molecules for biosensing [78].

### 3.4. Influence of Nanoparticles on the Biological Detection of Nematic Liquid Ccrystals

**Figure 9:**
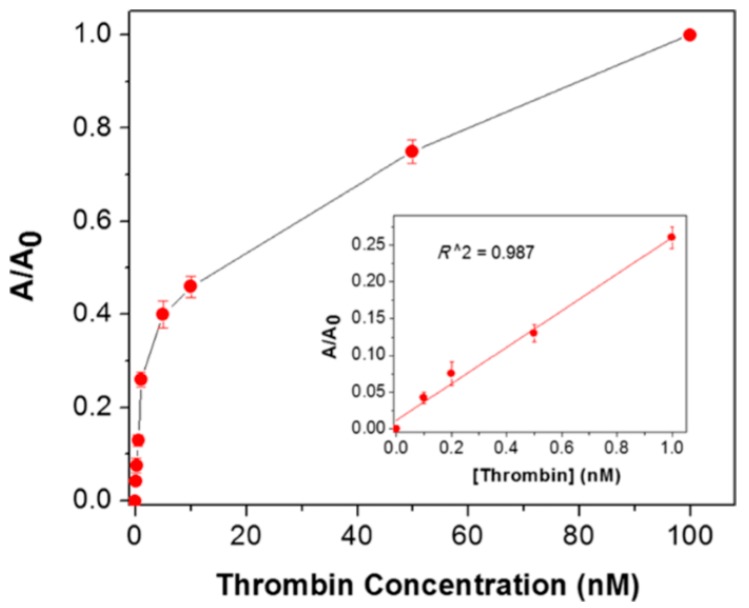
Correlations between the area ratio of the bright LC regions to the whole image and the concentration of thrombin. Inset: Linear relationship between the area ratio and the thrombin concentration. A = area of the bright LC regions. A_0_ = area of the whole image. The error bars represent the standard deviation of four measurements of a sample for each assay. Reproduced with permission from Ref. [70]. (American Chemical Society).

Recently in 2015, the use of spherical silver nanoparticle-doped LC biosensors have shown the potential to detect the biomolecule thrombin as shown in Figure 9 [70]. The GNPs along with the NLC are used to detect the acetylcholinesterase inhibitor and amplified detection of acetylcholine [48]. The key lies in the induced change in the alignment of the NLC sample cell for a particular interaction of the NLC molecule and target molecules. However, the biomolecules have also been detected by using the polyelectrolyte and a phospholipid monolayer at the aqueous/LC interface utilizing the same concept of reorientation of NLC molecules at the interface of NLC and aqueous solution [95].

**Figure 10:**
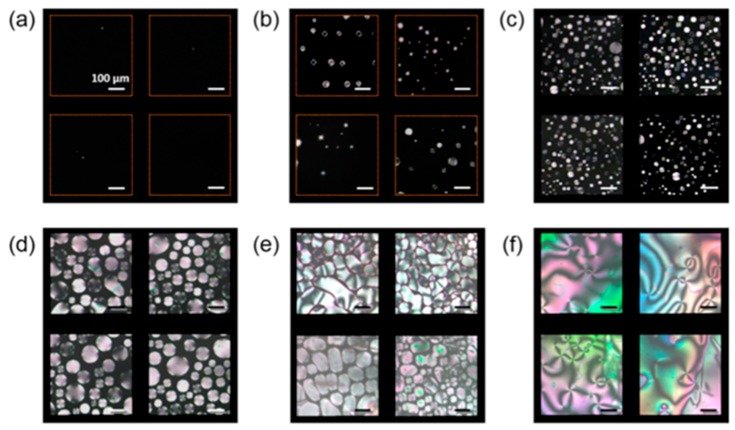
Polarized optical microscopic images of 4′-pentyl-4-biphenylcarbonitrile (5CB) doped with 0.01 wt % NiNSs (nickel nanospheres) in LC cells with substrates assembled with thrombin and the functionalized Au NPs. Thrombin is at concentrations of (**a**) 0.0, (**b**) 0.1, (**c**) 1, (**d**) 10, (**e**) 50, and (**f**) 100 nM. The scale bars in all panels are 100 μm. Here the GNP is attached with immobilized antithrombin aptamer on GPTMS film [(3-glycidoxypropyl)-trimethoxysilane which is an epoxy-group-rich compound], then the nickel nanoparticles doped 5CB NLC is introduced into the sample cell. Reproduced with permission from Ref. [70]. (American Chemical Society).

Nickel nanoparticles are used to compensate for the requirement of an alignment layer, where detection could be made without using any polymeric alignment layer, as nickel nanoparticles have been reported to induce uniform homeotropic alignment of NLC molecules (Figure 10) [70]. A sandwich system of nickel aptamer/thrombin is fabricated to get vertical alignment, and then the functionalized gold nanoparticles are used to produce the detection signal of planar alignment by disruption of NLC molecules when functionalized gold nanoparticles interact with thrombin. This has been found to be almost linear from a 10 to 100 nM concentration of thrombin. However, the large slope has also been observed at lower concentrations, with a transition between the two slopes resulting in the two stages of detection, which should further be explored.

**Figure 11:**
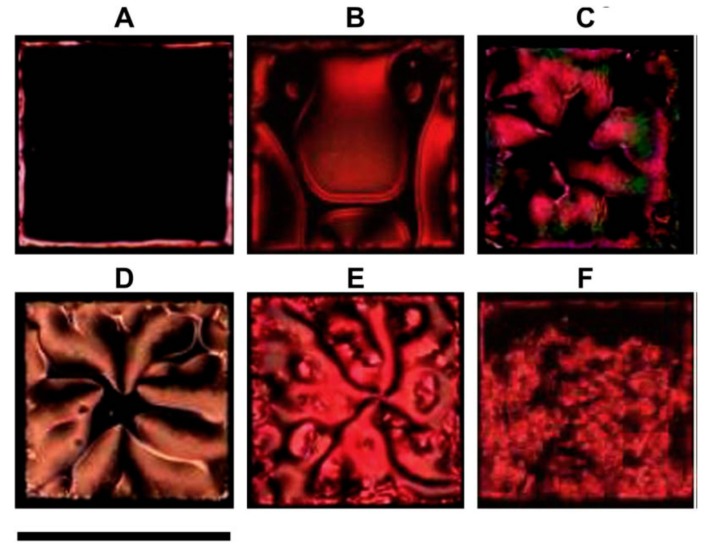
Interaction of a l-DLPC monolayer self-assembled at the aqueous-LC interface with solutions containing protein-coated Au NPs. Optical images of 5CB (crossed polarizers) captured (**A**) within 5 min after immersion of l-DLPC monolayer into AuNPs, (**B**) after 40 h contact of the l-DLPC with 50 nM of BSA-coated Au NPs, (**C**) after 60 h contact of the l-DLPC with 20 nM of BSA-coated Au NPs, (**D**) after 90 h contact of the l-DLPC with 2 nM of BSA-coated Au NPs, (**E**) after 32 h contact of the l-DLPC with 50 nM of neutravidin-coated Au NPs, and (**F**) after 2 h contact of the l-DLPC with 50 nM of fibrinogen-coated Au NPs. Scale bar = 283 mm. Reproduced with permission from Ref. [51]. (Elsevier).

Furthermore, protein-coated sodium citrate-stabilized gold nanoparticles are found to interact with a phospholipid (l-α-dilauroyl phosphatidylcholine, l-DLPC) layer, which in turn disrupts the NLC molecular alignment. This is observed in the optical imaging of the sample cell under the the crossed polarizers of optical polarizing microscopic transmission mode [51]. The optical texture formation of NLC (5CB) is found to depend on two parameters: one is the time of phospholipid monolayer on the glass, and the other is the concentration of l-DLPC. This is very obvious where that in both cases the interaction sites between gold nanoparticles and l-DLPC are supposed to offer a varying location of interaction to NLC in terms of molecular affinity, which allows the molecules to change their orientation randomly, so it is natural to get the change in optical contrast of the sample cell. The concept is again the reorientation of the NLC molecular director which induces the variation in the optical transmission and colors according to the change in birefringence, as illustrated in Figure 11.

When working on biosenisng applications, it is very selective to fabricate a particular device for a particular target. It is important to understand and utilize the elastic dipolar defect around the NPs for the sensing purposes [78]. The elastic deformation around the NPs could be the key mediation for the interaction between NPs. Other various defects in the NLC formed by NPs could be explored for binding the biological entity and be used for the biosensings. Research on NP-based biosensing applications is in the infant stage and needs to be explored further for conducting and non-conducting NPs of various shapes and sizes. It is most important to understand the interactions among the three candidates: biomolecules, NLCs, and NPs. If the self-assembly of the NPs after certain treatment (e.g., thermal treatment) could be controlled and could generate a periodic pattern [79], then it would lead to a photonic structure for guiding the electromagnetic waves, which is suitable for communication devices.

## 4. Conclusions

We have observed that nanoparticles are a property enhancer for NLCs due to the increment in the local orientation order parameter in the vicinity of nanoparticles. In the case of metal nanoparticles, the surface plasmon-like properties and the desired mediating molecules attached to the surface are the key points for the interaction with an NLC and its further application to biosensors. However, the origin of the biomolecular interaction is still unclear, and both theoretical quantification of parameters and experimental design need to be explored for the required device. We have discussed recent trends in the liquid crystal research arena and the development of metal NPs mediated NLC based biosensors. It has been found that the use of metal nanoparticles as dopants in an NLC allows the NLC to detect the presence of biomolecules.
